# Combined proteomics and metabolomics analysis reveal the effect of a training course on the immune function of Chinese elite short‐track speed skaters

**DOI:** 10.1002/iid3.70030

**Published:** 2024-10-01

**Authors:** Tieying Li, Jing Shao, Nan An, Yashan Chang, Yishi Xia, Qi Han, Fenglin Zhu

**Affiliations:** ^1^ Sports Nutrition Center, National Institute of Sports Medicine Beijing China; ^2^ Key Lab of Sports Nutrition State General Administration of Sport of China Beijing China; ^3^ National Testing & Research Center for Sports Nutrition, Ministry of Science and Technology of the People's Republic of China Beijing China; ^4^ School of Sport Medicine and Rehabilitation Beijing Sport University Beijing China

**Keywords:** immune, metabolomics, proteomics, short‐track speed skating

## Abstract

**Introduction:**

The aim of this study was to combine proteomics and metabolomics to evaluate the immune system of short‐track speed skaters (STSS) before and after a training course. Our research focused on changes in urinary proteins and metabolites that have the potential to serve as indicators for training load.

**Methods:**

Urine samples were collected from 21 elite STSS (13 male and 8 female) of the China National Team before and immediately after one training course. First‐beat sports sensor was used to monitor the training load. Proteomic detection was performed using a Thermo UltiMate 3000 ultra high performence chromatography nano liquid chromatograph and an Orbitrap Exploris 480 mass spectrometer. MSstats (R package) was used for the statistical evaluation of significant differences in proteins from the samples. Two filtration criteria (fold change [FC] > 2 and *p* < 0.05) were used to identify the differential expressed proteins. The Kyoto Encyclopedia of Genes and Genomes enrichment analysis for differential proteins was performed to identify the pathways involved. Nontargeted metabolomic detection was performed using ultra performance liquid chromatography tandem mass spectrometry (UPLC‐MS/MS_) with an ACQUITY 2D UPLC plus Q Exactive (QE) hybrid Quadrupole‐Orbitrap mass spectrometer. Differential metabolites were identified using non‐parametric statistical methods (Wilcox's rank test). Two filtration criteria (FC > 1.2 and *p* < 0.05) were used to identify differential metabolites. Combined analysis of proteomic and metabolomics were performed on the “Wu Kong” platform. Correlation analysis was performed using Spearman's rank correlation coefficient.

**Results:**

(1) The most upregulated proteins were immune‐related proteins, including complement proteins (C9, C4–B, and C9) and immunoglobulins (IgA, IgM, and IgG). The most downregulated proteins were osteopontin (OPN) and CD44 in urine. The correlation analysis showed that the content of OPN and CD44 (the receptor for OPN) in urine were significantly negatively correlated with the upregulated immune‐related proteins. The content of OPN and CD44 is sex‐dependent and negatively correlated with the training load. (2) The most upregulated metabolites included lactate, cortisol, inosine, glutamine, argininosuccinate (the precursor for arginine synthesis), 3‐methyl‐2‐oxobutyrate (the catabolite of valine), 3‐methyl‐2‐oxovalerate (the catabolite of isoleucine), and 4‐methyl‐2‐oxopentanoate (the catabolite of leucine), which is sex‐dependent and negatively correlated with OPN and CD44. (3) The joint analysis revealed five main related pathways, including the immune and innate immune systems. The enriched immune‐related proteins included complements, immunoglobulins, and protein catabolism‐related proteins. The enriched immune‐related metabolites included cAMP, N‐acetylgalactosamine, and glutamate. (4) There is a significant negative correlation between the content of OPN and CD44 in urine and the training load.

**Conclusion:**

One training course can lead to the activation of the immune system and a sex‐dependent decrease in the content of OPN and CD44. Training load has a significant and negative correlation with the content of OPN and CD44, suggesting that OPN and CD44 could be potential indicators for training load.

## Graphical abstract

One training course stimulates the immune response, and this reaction can be significantly reflected in urine. Moreover, the osteopontin (OPN)/CD44 pathway may be a potential indicator for training load in elite short track speed skaters.

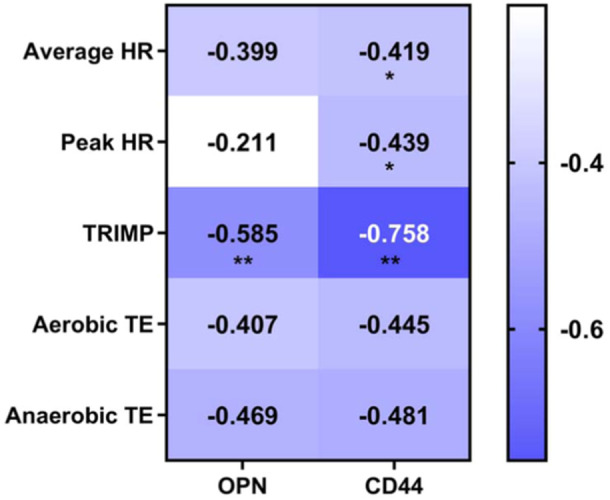

## 1 INTRODUCTION

One bout of exercise can trigger complex physiological and biochemical responses, including changes in gene transcription, protein expression, metabolic pathways, and inflammatory markers, among others.^1^ The immune system is highly responsive to both acute and chronic exercise training, as demonstrated by several decades of research in exercise immunology.^2^ The “open window” theory is characterized by short‐term immune system suppression, lasting for 3−72 h, following an acute bout of endurance exercise.^3^, ^4^ After stressful exercise, the blood can have a change in protein content, which includes complement, C‐reactive, transport, anti‐proteases, coagulation, and fibrinolytic proteins.^5^ Exercise‐associated immune cells can be redistributed into the circulation based on the intensity, duration, and volume of exercise.^6^, ^7^, ^8^, ^9^

Rapid developments in mass spectrometry, genomics,^10^ proteomics,^11^ and metabolomics^12^ technologies have provided new insights and tools to reveal the response of the immune system to acute and chronic exercise.^2^, ^13^, ^14^, ^15^ Proteomic and metabolomic studies have identified molecular changes in complement activation,^16^ immunoglobulins,^17^ immunocyte molecules,^18^ and acute inflammatory response‐related metabolic pathways.^19^ Sixty‐five older people aged 65−85 years were subjected to a combination of a high‐protein diet (17 weeks) and strength training (twice per week for the last 7 weeks), and the plasma samples collected before and after the intervention were assessed using proteomics. The high‐protein diet had a significant impact on 14 proteins, which include those involved in the innate immune system, lipid transport, and blood coagulation. However, further changes were not elicited by additional strength training.^20^ Combined analysis of proteomics and metabolomics of the urine samples before and after high intensity interval training (HIIT) exercise in 23 healthy young soccer players. The results revealed the effects of HIIT on energy metabolism, immune response, and physiological oxidative stress processes.^21^

Professional athletes may experience inflammation and immune function decline due to overtraining and insufficient recovery, which can even impair their sports performance.^22^ Suitable, reliable, noninvasive, and time‐efficient methods are required to investigate fundamental indicators, such as monitoring prospectively fatigue, injury, and nutritional supplements. The omics technology provides new possibilities for training monitoring. Short‐track speed skating competition covers distances of 500, 1000, and 1500 m, with a duration of approximately 40 s to 2.5 min, and require a maximal intensity at speeds beyond maximal oxygen uptake (VO_2max_), world‐class short‐track speed skaters (STSS) can be developed in speed, hybrid, and endurance profiles.^23^ The research of this sport focus on sport injury and technical‐tactics analysis, there is a lack of research on the physiological responses of STSS to acute exercise. Therefore, we combined the proteomics and metabolomics to analyze the urine samples of STSS athletes before and after a training session. Our goal is to analyze the modifications in physiological indicators in urine prior and afterwards of a training course to propose individualized suggestions for daily training and postexercise recovery of STSS.

## 2 MATERIAL AND METHODS

### 2.1 Participants

The study participants included 13 male and 8 female elite Chinese STSS. Anthropometric data were collected (Table 1). Urea samples were collected before (Pre group) and immediately after (Post group) a training course on Monday morning. Furthermore, to explore the differences between male and female, data is divided into four subgroups (Pre‐Male, Pre‐Female, Post‐Male and Post‐Female group). This study was approved by the Ethics Committee of the Institute of Sports Medicine of the General Administration of Sports of China (approval no. 201904).

**Table 1:** Anthropometric parameters (mean ± SD).

Index	Male (*n* = 13)	Female (*n* = 8)
Age (years)	22.15 ± 3.67	23.50 ± 3.30
Height (cm)	177.08 ± 6.43	169.75 ± 4.30
Body weight (kg)	72.03 ± 6.41	61.13 ± 2.07
Body fat percentage (%)	12.54 ± 1.14	20.26 ± 3.10
Fat weight (kg)	8.67 ± 1.12	11.54 ± 1.33
Muscle weight(kg)	60.41 ± 5.31	46.69 ± 2.62
BMI (Kg/m^2^)	22.15 ± 3.67	23.50 ± 3.30
Abdominal fat (%)	22.85 ± 1.30	21.23 ± 0.93
Abdominal fat (%)	10.98 ± 3.38	17.10 ± 4.10
Hip fat (%)	10.36 ± 1.58	21.20 ± 3.24
Abdominal to hip fat ratio	1.06 ± 0.28	0.82 ± 0.12
Basal metabolic rate (kcal)	1764.47 ± 136.48	1437.03 ± 33.37
Bone mineral (kg)	2.94 ± 0.35	2.60 ± 0.17

### 2.2 Training load

**Figure 1:**
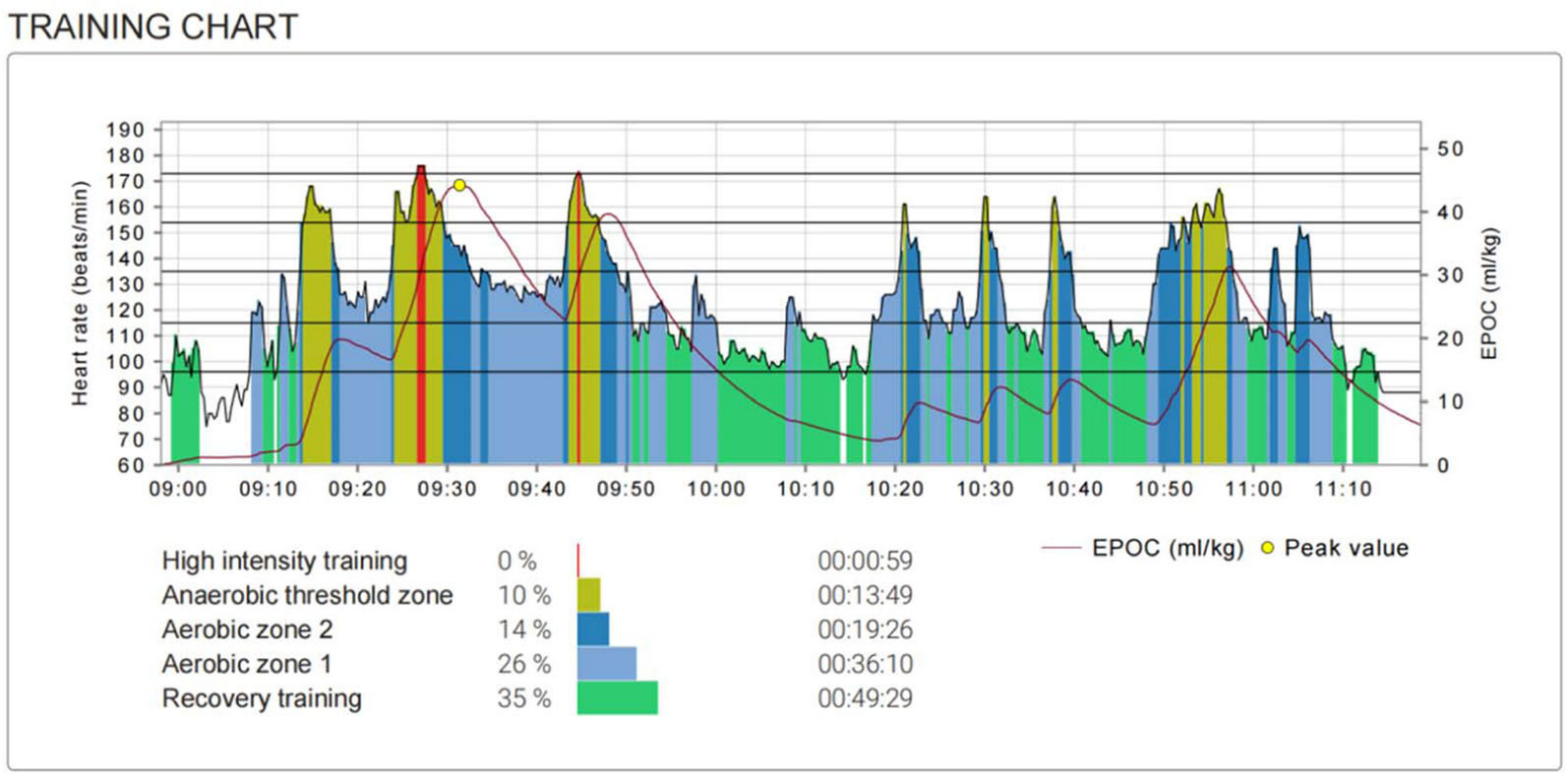
Heart rate variation chart of an athlete during training course.

Training class content (1) On‐ice, two sets of speed increase training: two laps and seven laps, three sets of speed training: one laps of a constant speed plus 1.5 laps of sudden acceleration training. Off‐ice, six sets of squats and standing broad jumps training, follow up with a 20 min low to moderate intensity run. Athletes are free to drink water during the course. Here is the heart rate variation chart of an athlete during the traning course (Figure 1).

The Firstbeat Sport Sensor (Firstbeat Technologies Oy) was used to monitor the training load and record the daily energy expenditure. The training impulse (TRIMP) is a measure used to quantify the training load accumulated during a course. TRIMP considers exercise intensity as calculated using the heart rate (HR) reserve method and the exercise duration.^24^ Training effects including the aerobic and anaerobic training effects, which were also calculated using HR reserve. The anaerobic training effect indicates the magnitude of the impact of a single training course on the ability to repeat high‐intensity sprints. The greater the impact of training activities on anaerobic metabolism, the higher the TE value, which is related to the training interval time, acceleration, and recovery level before the interval begins. The anaerobic training effect indicates the degree of impact of one training course on the development of VO_2max_. The greater the impact of training activities on aerobic metabolism, the higher the TE value. According to the technical reference data provided by the instrument manufacturer, TRIMP value is 70−140 indicating moderate training intensity, aerobic training effect value is 2.0−2.9 indicating training intensity is beneficial for maintaining aerobic capacity, anaerobic training effect value is 2.0−2.9 indicating training intensity is beneficial for maintaining anaerobic capacity. Table 2 lists the average energy expenditure and TRIMP values during the training.

**Table 2:** The training loads (mean ± SD).

Index	
Energy Expenditure	592.25 ± 150.03
TRIMP	101.25 ± 26.22
HRmax	180.00 ± 4.81
Average HR	118.00 ± 7.97
Training effect: Aerobic	2.56 ± 0.31
Training effect: Anaerobic	2.29 ± 0.21

### 2.3 Urine collection

Urine samples were collected before and immediately after the training course. Midstream urine was collected by the participants following the instructions. Collected urine samples were treated immediately in the lab with sodium azide (final concentration of 0.1%) to arrest bacterial growth and centrifuged at 2000*g* for 10 min at 4°C to remove sediments. Aliquots (5 mL) were stored at −80°C before metabolomic analysis.

### 2.4 Urine proteomics

#### 2.4.1 Sample preparation

Protein concentration was determined using the Bradford protein assay kit (Bio‐Rad). Subsequently, the proteins were reduced by dithiothreitol at 37°C in a water bath for 30 min and alkylated by iodoacetamide at room temperature (26°C) for 30 min in the darkroom. The proteins were then digested with trypsin (Promega) following (filter‐aided sample preparation) manufacturer protocol.

#### 2.4.2 LC‐MS/MS analysis

(1) Data Independent Acquisition (DIA) quantification (Nano‐LC‐MS/MS)

The dried peptide samples were reconstituted with mobile phase A (2% acetonitrile (ACN), 0.1% formic acid (FA)) and centrifuged at 20,000*g* for 10 min, and the supernatant was collected for injection. The separation was performed using a Thermo UltiMate 3000 ultra high performence chromatography. The sample was first enriched in the trap column and desalted. Thereafter, the sample was entered into a tandem self‐packed C18 column (150 μm internal diameter, 1.8 μm column size, 35 cm column length) and separated at a flow rate of 500 nL/min via the following effective gradient: 0−5 min, 5% mobile phase B (98% ACN, 0.1% FA); 5−90 min, mobile phase B linearly increased from 5% to 25%; 90−100 min, mobile phase B increased from 25% to 35%; 100−108 min, mobile phase B increased from 35% to 80%; 108−113 min, 80% mobile phase B; 113.5−120 min, 5% mobile phase B.

(2) DIA Mass spectrometry detection

The peptides separated by liquid phase chromatography were ionized by a nano electron spray ioniztion (ESI) source and then passed to a tandem mass spectrometer Oritrap Exploris 480 (Thermo Fisher Scientific) for DIA (Data Independent Acquisition) mode detection. The main parameters were set: ion source voltage was set to 1.9 kV, MS1 mass spectrometer scanning range was 400−1250 m/z; resolution was set to 120,000; maximal injection time (MIT) 90 ms; 400−1250 m/z was equally divided to 50 continuous windows MS/MS scan. MS/MS collision type HCD, collision energy NCE 30; MIT was auto mode. Fragment ions were scanned in Orbitrap, MS/MS resolution 30,000. AGC was: MS 300%, MS/MS 1000%.

#### 2.4.3 DIA data analysis

The DIA data was analyzed using the iRT peptides for retention time calibration. Then, based on the target‐decoy model applicable to SWATH‐MS, false positive control was performed with false discovery rate (FDR) 1%, therefore obtaining significant quantitative results. Differential analysis was performed by MSstats^25^ an R package from the Bioconductor repository. Differential protein screening was performed based on the fold change (FC) > 2 and *p* < 0.05 (identified using *t*‐tests) as the criterion for the significant difference. Biological pathway analysis was performed through metabolite set enrichment analysis using the BGI Dr. Tom multiomics data mining system,^26^ which is a suite under the Kyoto Encyclopedia of Genes and Genomes (KEGG) database.

### 2.5 Urine metabolomics

#### 2.5.1 Untargeted UPLC–MS/MS analysis

UPLC (ACQUITY 2D, Waters) and Q Exactive (QE) hybrid Quadrupole‐Orbitrap mass spectrometer (Thermo Fisher Scientific) were used for untargeted metabolomics analysis. The QE mass spectrometer was operated at a mass resolution of 35,000 and a scan range of 70−1000 m/z. In the ultra performance liquid chromatography tandem mass spectrometry (UPLC‐MS/MS) method, QE was operated in positive or negative ESI mode, and a C18 reverse‐phase UPLC column was used (UPLC BEH C18, 2.1 × 100 mm, 1.7 µm; Waters).

#### 2.5.2 Compound identification and quantification

The identification of metabolites in samples requires strict matching of three criteria between experimental data and library entry: narrow window retention index (RI), accurate mass with a variation of less than 10 ppm, and MS/MS spectra with high forward and reverse searching scores. The peak area for each metabolite was calculated from the area under the curve.

#### 2.5.3 Data analysis

For certain errors that may cause differences in the initial sample size of each sample, normalization is performed using urine creatinine to ensure that each sample does not have erroneous interpretations of metabolic level differences due to differences in sample size.

For each metabolite, the values in Peak Area Data were first subjected to Log2 logarithmic conversion. Then divide by the median value arranged in all samples run daily after logarithmic conversion, to make the median becomes 1. Finally, fill in the missing values with the minimum value in the original data. Welch *t*‐tests were used for initial analyses. The FDR was used for multiple test correction. Parametric data are presented as means ± standard deviation (SD). Principal component analysis (PCA) were conducted to investigate differences in polar and nonpolar metabolite profiles in urine samples. Metabolites with an FDR < 0.05 were considered significant. On the significant metabolites, as identified by FDR‐adjusted analysis of variance, a pair‐wise urine metabolite relative intensity FC was calculated between groups. The pairwise FC satisfying the criteria of Log_2_FC > ± 1 (equivalent to FC > 2) and *p* < 0.05 (identified using *t*‐tests) was considered significant. Pathway enrichment analysis was conducted using MetPA^27^ based on the KEGG database and Pathview.^28^ Only the significantly altered metabolites with associated KEGG ID were included. A significance analysis of pathway enrichment was performed using a hypergeometric test. All statistical analyses were performed using the R language. Combined analysis of the proteomic and metabolomic was performed on the “Wu Kong” platform (http://www.omicsolution.com/wkomics/wkold).^29^

### 2.6 Correlation analysis

Correlation analysis was conducted using the statistical package for the social sciences 17.0 software and assessed using the Spearman's rank correlation coefficient. Statistical significance was defined as *p* < 0.05. Data were presented as the mean ± SD. The correlation coefficient, denoted as *r*, describes the strength of the correlation between two variables. The larger the absolute value of *r*, the stronger the correlation. The value of *r* is between −1 and +1. If *r* > 0, it indicates that the two variables are positively correlated, meaning that the larger the value of one variable, the larger the value of the other variable. If *r* < 0, it indicates that the two variables are negatively correlated, meaning that the larger the value of one variable, the smaller the value of the other variable. The larger the absolute value of *r*, the stronger the correlation.

## 3 RESULTS

### 3.1 Urine proteomics analysis

#### 3.1.1 Urine proteomics overview

**Figure 2:**
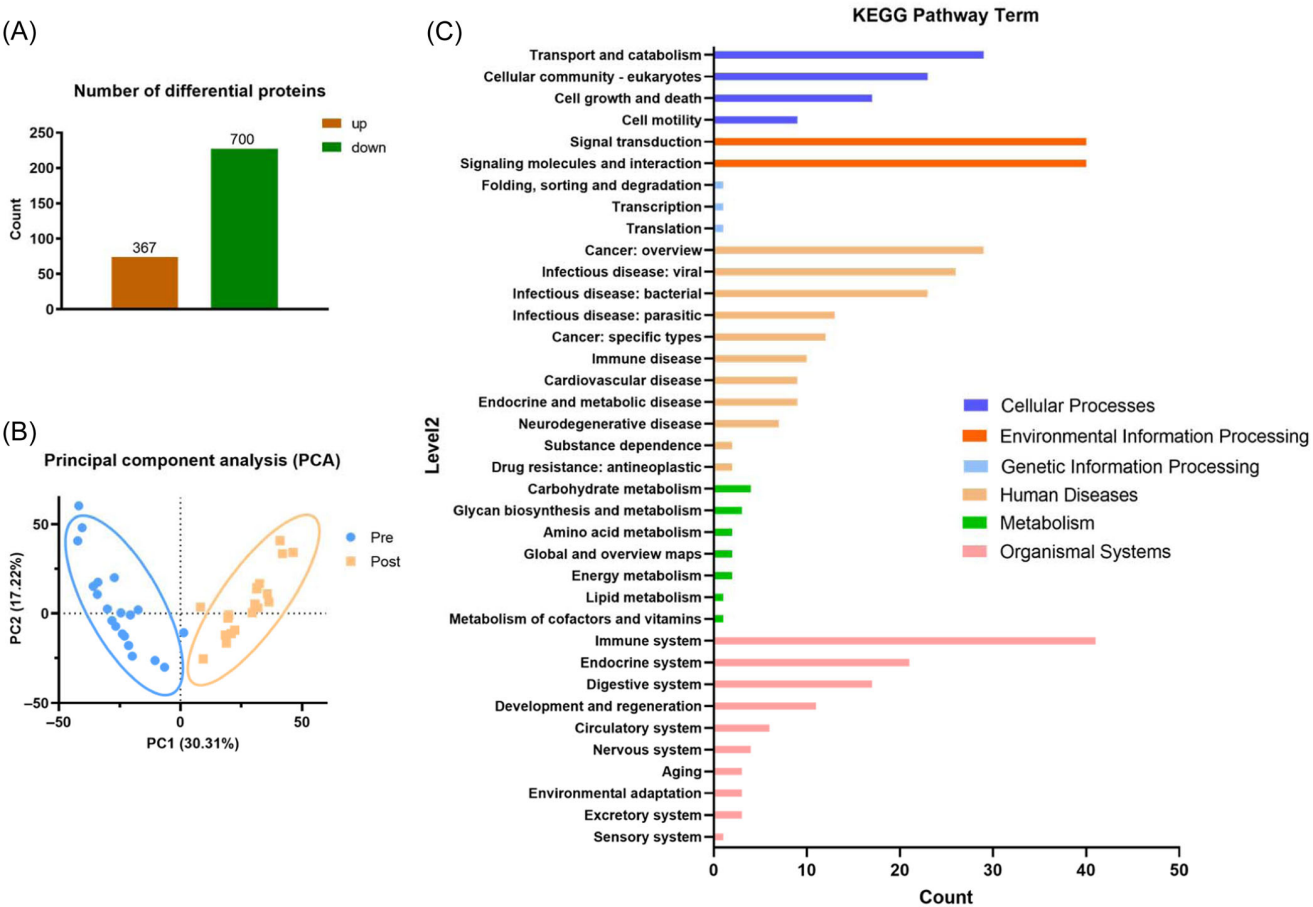
Proteomics overview. (A) Number of differential proteins, up‐(red) and down‐(green) regulated. (B) Principal component analysis for identified differential metabolites between Pre (blue) and Post (yellow) group. The X‐axis is the first principal component, and the Y‐axis is the second principal component. (C) KEGG pathway enrichment analysis of all differential proteins identified, the X‐axis represents the number of proteins annotated to a certain KEGG pathway category, and the Y‐axis represents the KEGG pathway category. KEGG, Kyoto Encyclopedia of Genes and Genomes.

There is 1067 differential proteins (FC > 2 and *p* < 0.05) were identified after a training course, in which 367 proteins significant up regulated and 700 proteins down regulated (Figure 2A). PCA analysis were performed on the proteomics data, results showed that proteins identified between Pre and Post group were clustered into two discriminating groups in score plots of PCA, indicating that proteins in the urine were significantly influenced by a training course (Figure 2B). KEGG pathway enrichment were shown in Figure 2C. Six main classes of functions with different colors were shown. Proteins related to the immune system, endocrine system, signal transduction and metabolism, which may crucially be involved in the process of exercise and recovery.

#### 3.1.2 KEGG pathway enrichment of differential proteins between subgroups

**Figure 3:**
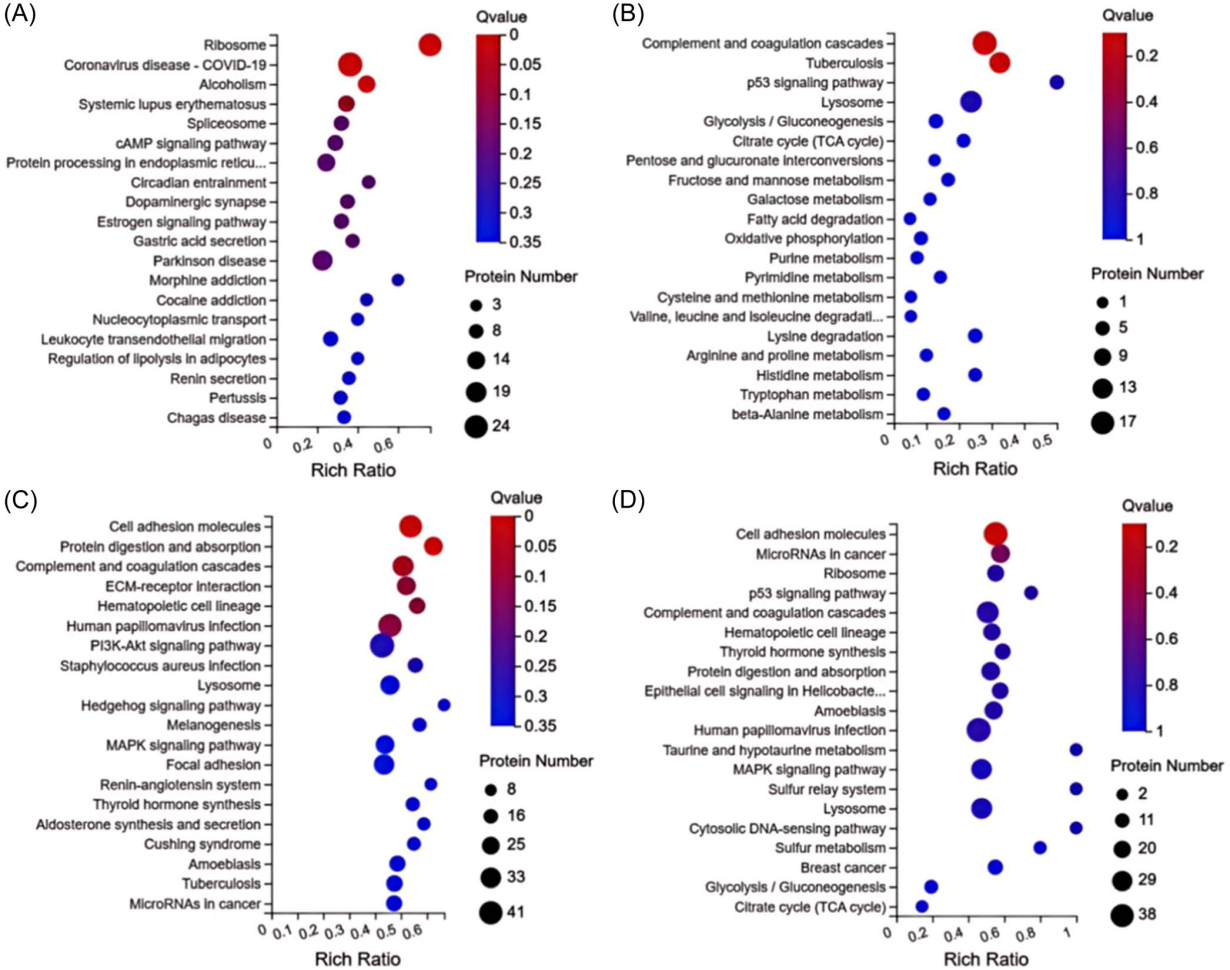
Enriched proteins sets of the KEGG pathway. (A−D) The KEGG pathway enrichment analysis of the differential proteins between the Pre‐Male group and Pre‐Female group, between the Post‐Male group and Post‐Female group, Post‐Male group and Pre‐Male group, between the Post‐Female group and Pre‐Female group. The X‐axis represents the enrichment ratio (the ratio of the number of proteins annotated to a certain pathway in the selected protein set to the number of proteins annotated to that pathway in the total protein set of the species, calculated as Rich Ratio = Term Candidate Protein Number/Term Protein Number), the Y‐axis represents the KEGG pathway, and the size of bubbles indicates the number of proteins annotated to a certain KEGG pathway. The color represents the enrichment significance value (*p* Value, see legend for details), and the redder the color, the smaller the significance value. KEGG, Kyoto Encyclopedia of Genes and Genomes.

By the KEGG pathway enrichment, we found that except the Pre‐Male/Pre‐Female group (Figure 3A), the other three comparison groups were enriched in the complement pathway in the top 5 pathway, especially between the Post‐Male group and Post‐Female group (Figure 3B).

#### 3.1.3 Regulated proteins of the immune system

**Figure 4:**
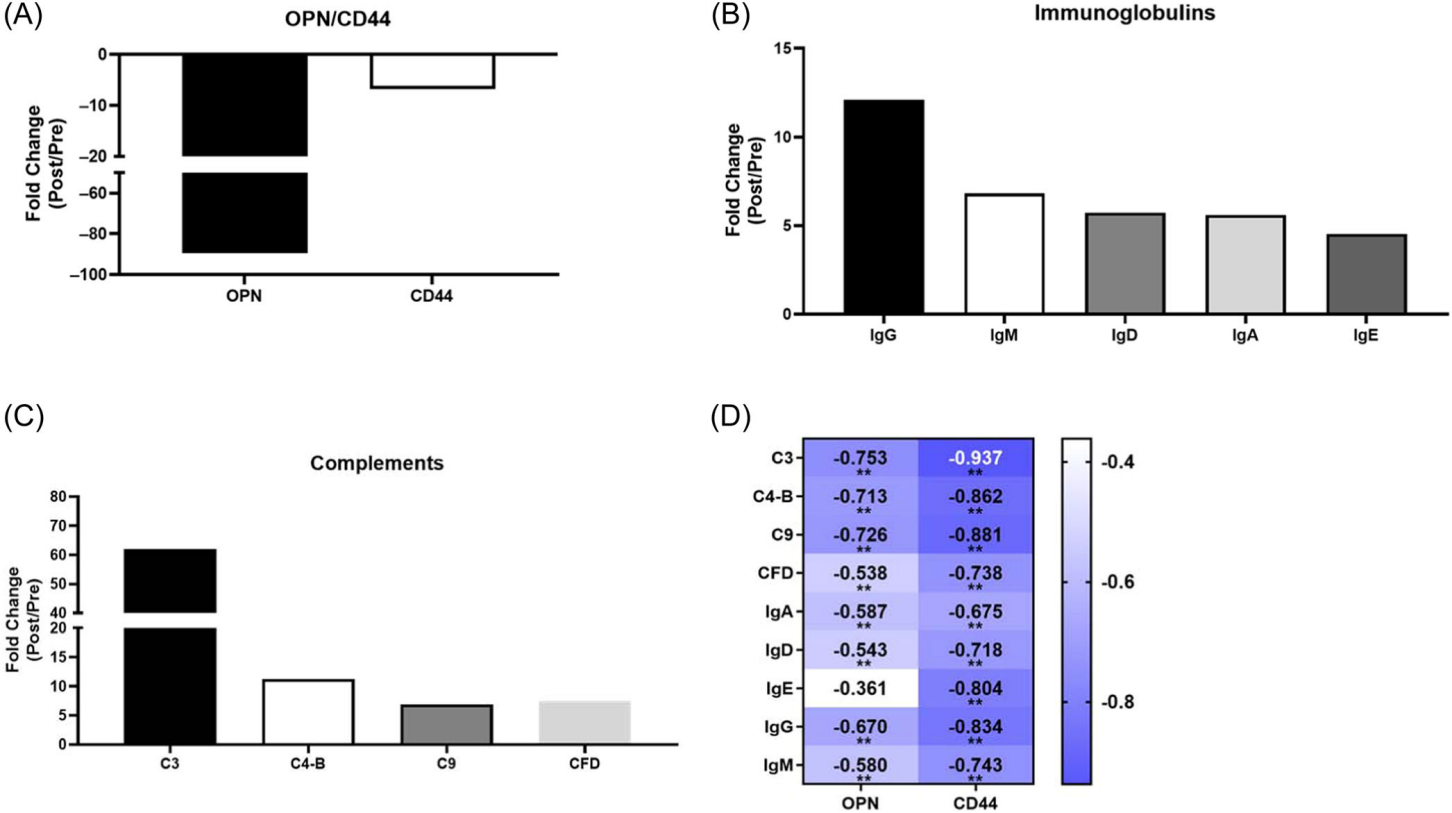
The most differential proteins between the Pre and Postgroup. (A) The fold change of OPN and its receptor CD44. (B) The most upregulated immunoglobulins. (C) The most upregulated proteins of the complement system. (D) The correlation analysis between the content of OPN/CD44 and regulated immune‐related proteins in urine (**p＜0.01). C3, complement 3; C4‐B, complement C4‐B; C9, component 9; CFD, complement factor D; OPN, osteopontin.

Osteopontin (OPN) is the most downregulated protein of the immune system with 90 FC, and CD44 with nearly 10 FC is the receptor of OPN (Figure 4A). The most upregulated immunoglobulins is IgA with more than 10 FC, IgD, IgG, and IgM with more than fiveFC (Figure 4B). The most upregulated proteins of the complement system is complement 3 (C3) with more than 60 FC, and complement C4‐B (C4‐B), component 9 (C9), and complement factor D (CFD) with more fiveFC (Figure 4C). The correlation analysis of the content of OPN/CD44 in urine and regulated immune‐related proteins in urine (**p＜0.01) (Figure 4D).

#### 3.1.4 Regulated proteins of the immune system between subgroups

Table 3 shows the normalized raw data of each subgroups. The normalized peak areas were log‐transformed (log2) to reduce the skewness of the data distribution and obtain an approximately normal distribution. When calculating the difference between groups, it should be subtracting two sets of data and then calculating the power function of 2.

**Table 3:** The log‐transformed normalized peak areas of differential proteins (mean ± SD).

Protein	Pre			Post		
	Total (*n* = 21)	Male (*n* = 13)	Female (*n* = 8)	Total (*n* = 21)	Male (*n* = 13)	Female (*n* = 8)
OPN	27.87 ± 1.01	28.08 ± 0.88	27.51 ± 1.16	21.38 ± 3.04	20.38 ± 3.45	22.76 ± 1.70
CD44	25.83 ± 0.81	25.83 ± 0.86	25.83 ± 0.78	22.96 ± 0.97	23.34 ± 1.02	22.44 ± 0.64
IgG	19.04 ± 2.24	18.70 ± 2.36	19.58 ± 2.05	22.64 ± 0.69	22.51 ± 0.58	22.81 ± 0.83
IgM	21.01 ± 1.91	20.64 ± 2.02	21.61 ± 1.66	23.78 ± 0.83	23.48 ± 0.65	24.19 ± 0.90
IgD	13.92 ± 0.79	13.61 ± 0.81	14.53 ± 0.02	16.31 ± 1.56	15.90 ± 1.69	16.82 ± 1.30
IgA	20.01 ± 2.53	19.90 ± 2.76	20.19 ± 2.28	22.50 ± 0.85	22.66 ± 0.84	22.27 ± 0.85
IgE	14.49 ± 0.65	14.63 ± 0.83	14.35 ± 0.71	16.67 ± 0.90	16.22 ± 0.85	17.23 ± 0.63
C3	15.10 ± 2.08	15.27 ± 2.10	14.83 ± 2.16	21.05 ± 2.11	20.07 ± 2.22	22.41 ± 0.88
C4‐B	15.45 ± 1.25	15.36 ± 1.21	15.59 ± 1.38	18.24 ± 1.43	17.63 ± 1.45	19.08 ± 0.92
C9	14.32 ± 1.32	14.71 ± 1.18	13.60 ± 1.36	17.22 ± 1.81	16.74 ± 2.18	17.89 ± 0.87
CFD	17.80 ± 2.06	17.82 ± 2.29	17.76 ± 1.76	21.29 ± 0.43	21.22 ± 0.53	21.37 ± 0.25

Abbreviations: CFD, complement factor D; OPN, osteopontin.

Table 4 shows the differences between the four subgroups, the differential screening was performed based on the FC > 2 and *p* < 0.05 (identified using *t*‐tests) as the criterion for the significant difference. Before exercise, the IgM and IgA is significantly different between the Pre‐Male and the Pre‐Female group. After exercise, the OPN, IgE, C3, C4‐B in the Post‐Male group is significantly lower than the Post‐Female group, while the CD44 is higher. Whether in the male or female sub groups, the trend of urine protein changes caused by exercise is the same.

**Table 4:** The inter group comparison of differential proteins.

Protein	Pre‐Male/Pre‐Female		Post‐Male/Post‐Female		Post‐Male/Pre‐Male		Post‐Female/Pre‐Female		Post‐Total/Pre‐Total	
	FC	*p* Value	FC	*p* Value	FC	*p* Value	FC	*p* Value	FC	*p* Value
OPN	1.67	0.29	0.23	0.00	0.04	0.00	0.004	0.00	0.01	0.00
CD44	1.06	0.82	1.96	0.02	0.16	0.00	0.10	0.00	0.14	0.00
IgG	0.81	0.65	0.81	0.65	12.68	0.00	9.18	0.00	12.10	0.00
IgM	0.38	0.02	0.65	0.33	7.15	0.02	6.03	0.00	6.83	0.00
IgD	0.76	0.10	0.66	0.34	5.28	0.15	6.62	0.15	5.74	0.00
IgA	4.25	0.04	1.66	0.29	7.62	0.13	5.07	0.07	5.60	0.00
IgE	1.24	0.72	0.54	0.04	3.31	0.04	7.57	0.21	4.53	0.00
C3	1.04	0.93	0.26	0.00	21.38	0.00	61.07	0.03	61.90	0.00
C4‐B	0.85	0.65	0.37	0.01	5.59	0.01	9.88	0.02	6.92	0.00
C9	0.43	0.11	0.59	0.31	6.64	0.00	10.17	0.09	7.49	0.00
CFD	1.73	0.37	1.00	1.00	4.90	0.00	8.10	0.00	11.2	0.00

Abbreviations: CFD, complement factor D; OPN, osteopontin.

### 3.2 Urine metabolomic analysis

#### 3.2.1 Metabolomics overview

**Figure 5:**
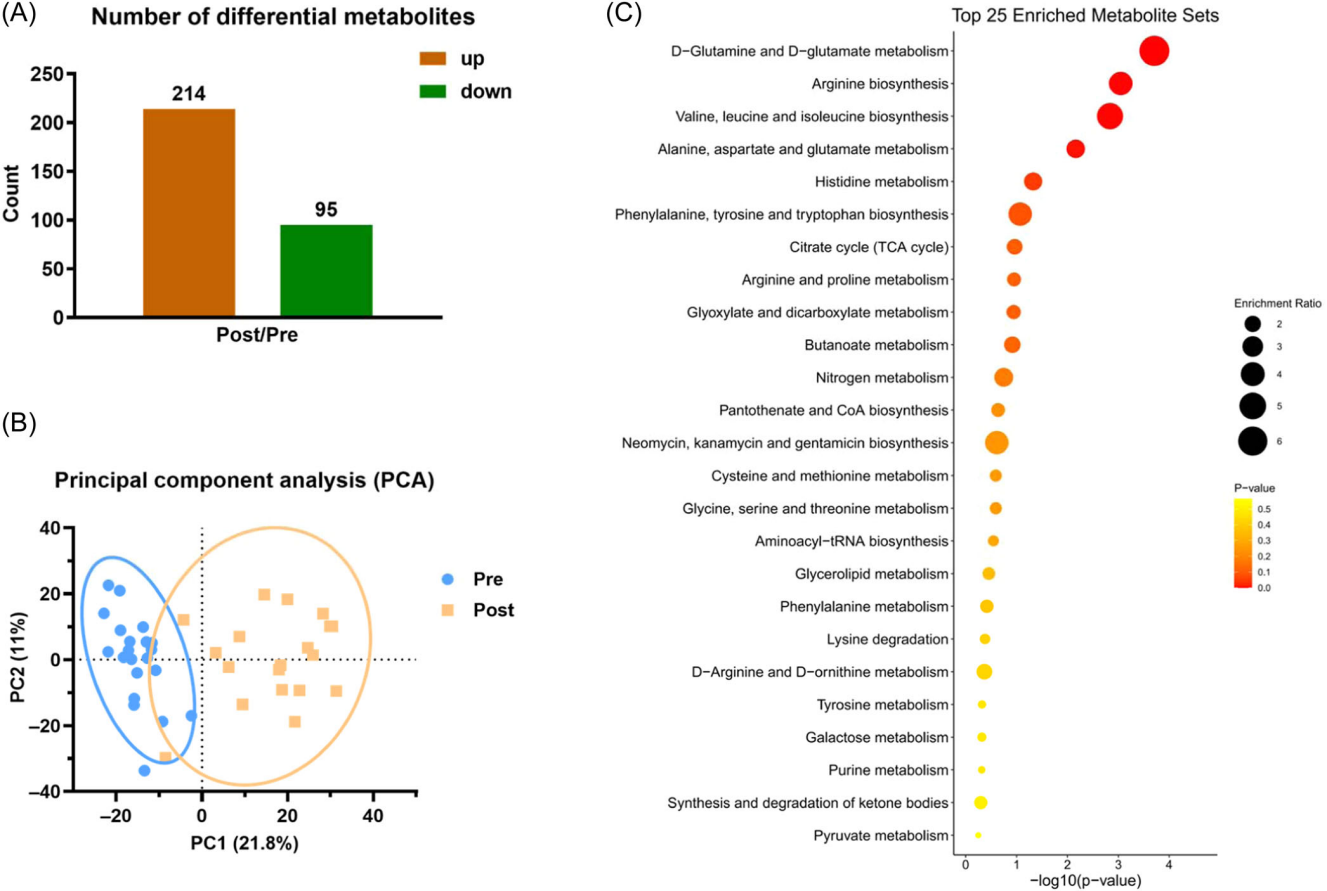
Metabolomics overview. (A) Number of differential metabolites, up (red) and down (green) regulated. (B) Principal component analysis of the Pregroup (blue) and the Postgroup (yellow); the X‐axis is the first principal component, and the Y‐axis is the second principal component. (C) Top 25 enriched metabolite sets of the KEGG pathway with all differential metabolites identified in the urine samples. The X‐axis is the enrichment ratio, and the Y‐axis is GO Term. The size of the bubble represents the number of differential metabolites annotated on a GO Term. The color represents the enrichment *p* Value. KEGG, Kyoto Encyclopedia of Genes and Genomes.

After a training course, 309 metabolites were significantly changed (FC > 2, *p* < 0.05) in which 214 metabolites were significantly upregulated and 95 metabolites were significantly downregulated (Figure 5A). PCA analysis was performed on the identified metabolites, results showed that metabolites identified were clustered into two discriminative groups in score plots of PCA, indicating that metabolites in the urine were significantly influenced by one training course (Figure 5B). To explore the function of differential metabolites, the metabolites with significant variations were analyzed by KEGG database. KEGG pathway enrichment were shown (Figure 5C).

#### 3.2.2 Enriched metabolite sets of the KEGG pathway of differential metabolites between subgroups

**Figure 6:**
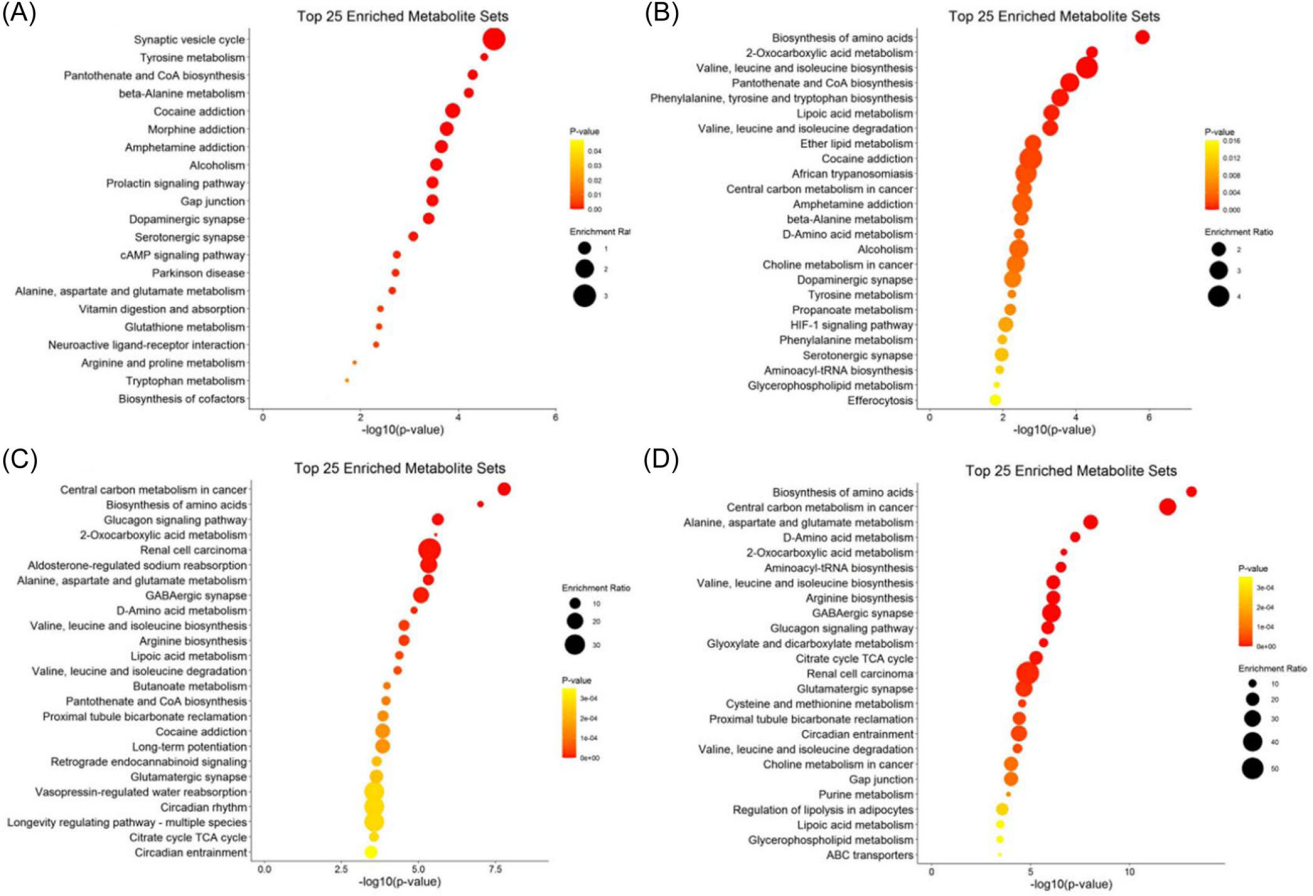
Top 25 enriched metabolite sets of the KEGG pathway with all differential metabolites identified in the subgroups in the urine samples. The X‐axis is the enrichment ratio, and the Y‐axis is GO Term. The size of the bubble represents the number of differential metabolites annotated on a GO Term. The color represents the enrichment *p* Value. KEGG, Kyoto Encyclopedia of Genes and Genomes.

The most enriched pathway of differential metabolites between the Pre‐Male group and Pre‐Female group is synaptic vesicle cycle, tyrosine metabolism and the pantothenate and CoA biosynthesis (Figure 6A). The most enriched pathway of differential metabolites between the Post‐Male group and Post‐Female group is biosynthesis of amino acids, 2‐oxocarboxylic acid metabolism and valine, leucine and isoleucine biosynthesis, all of them are closely related to the metabolism of branched chain amino acids (Figure 6B). The most enriched pathway of differential metabolites between the Post‐Male group and Pre‐Male group is central carbon metabolism in cancer, biosynthesis of amino acids and glucagon signaling pathway, all of them are closely related to energy recovery after exercise (Figure 6C). The most enriched pathway between the Post‐Female group and Pre‐Female group is biosynthesis of amino acids, central carbon metabolism in cancer and the Alanine, aspartate and glutamate metabolism (Figure 6D).

#### 3.2.3 Correlation analysis of OPN/CD44 and the most regulated metabolites

**Figure 7:**
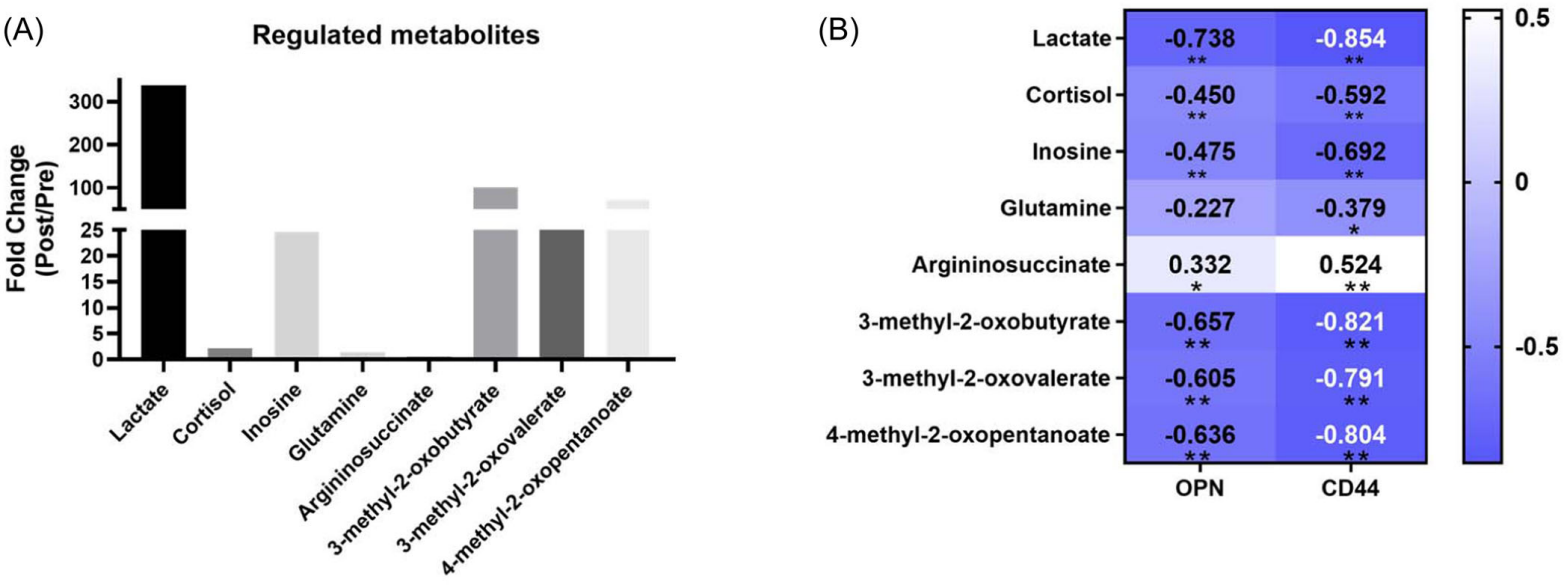
(A) The most regulated metabolites related to training monitoring. (B) The correlation analysis of the content of OPN/CD44 and the most regulated metabolites in urine. The color scale denotes the Spearman's rank correlation coefficient, and the significance levels in the correlation tests are denoted as **p < 0.01.

The most upregulated metabolites related to training monitoring including lactate, cortisol, inosine, glutamine, 3‐methyl‐2‐oxobutyrate (the catabolite of valine), 3‐methyl‐2‐oxovalerate (the catabolite of isoleucine) and 4‐methyl‐2‐oxopentanoate (the catabolite of leucine), while argininosuccinate (the precursor for arginine synthesis) significantly downregulated (Figure 7A). The most upregulated metabolites are lactate, 3‐methyl‐2‐oxobutyrate, and 4‐methyl‐2‐oxopentanoate with nearly 25 times increase.

The heatmap displays the significantly differential metabolites related to training monitoring between Pre and Postgroup with the content of OPN/CD44 (Figure 7B) in urine, in which lactate is the most strong negative correlated with the OPN/CD44.

#### 3.2.4 The most regulated metabolites in the subgroups

Table 5 shows the normalized raw data of each subgroups. Peak Area Data were first subjected to Log2 logarithmic conversion. Then divide by the median value arranged in all samples run daily after logarithmic conversion, to make the median becomes 1.

**Table 5:** The log‐transformed normalized peak areas of differential metabolites (mean ± SD).

Protein	Pre			Post		
	Total	Male	Female	Total	Male	Female
Lactate	0.75 ± 0.01	0.75 ± 0.01	0.76 ± 0.01	1.02 ± 0.05	1.00 ± 0.05	1.02 ± 0.05
Cortisol	0.95 ± 0.06	0.94 ± 0.06	0.97 ± 0.06	1.03 ± 0.05	1.01 ± 0.05	1.03 ± 0.05
Inosine	0.95 ± 0.05	0.94 ± 0.05	0.97 ± 0.05	1.13 ± 0.11	1.09 ± 0.12	1.13 ± 0.11
Glutamine	0.99 ± 0.02	1.00 ± 0.02	0.98 ± 0.01	1.01 ± 0.02	1.01 ± 0.02	1.01 ± 0.02
Argininosuccinate	1.01 ± 0.03	1.01 ± 0.03	1.02 ± 0.03	0.96 ± 0.05	0.98 ± 0.05	0.96 ± 0.05
3‐methyl‐2‐oxobutyrate	0.95 ± 0.03	0.95 ± 0.03	0.96 ± 0.03	1.23 ± 0.09	1.20 ± 0.09	1.23 ± 0.09
3‐methyl‐2‐oxovalerate	0.97 ± 0.03	0.96 ± 0.03	0.98 ± 0.02	1.14 ± 0.07	1.11 ± 0.07	1.14 ± 0.07
4‐methyl‐2‐oxopentanoate	0.97 ± 0.02	0.96 ± 0.02	0.97 ± 0.01	1.20 ± 0.08	1.17 ± 0.08	1.20 ± 0.08

Table 6 shows the difference between the four subgroups, the differential screening was performed based on the FC > 2 and *p* < 0.05 (identified using *t*‐tests) as the criterion for the significant difference. Before exercise, the glutamine is higher and the lactate is lower in the Pre‐Male group than the Pre‐Female group. After exercise, the lactate, cortisol, 3‐methyl‐2‐oxobutyrate, 3‐methyl‐2‐oxovalerate, 4‐methyl‐2‐oxopentanoate in the Post‐Male group is significantly lower than the Post‐Female group. Whether in the male or female sub group, the trend of the differential metabolites changes caused by exercise is the same.

**Table 6:** The inter group comparison of differential metabolites.

Metabolites	Pre‐Male/Pre‐Female		Post‐Male/Post‐Female		Post‐Male/Pre‐Male		Post‐Female/Pre‐Female		After‐Total/Pre‐Total/	
	FC	*p* Value	FC	*p* Value	FC	*p* Value	FC	*p* Value	FC	*p* Value
Lactate	0.63	0.01	0.41	0.00	301.66	0.00	466.82	0.00	337.81	0.00
Cortisol	0.46	0.06	0.62	0.04	2.66	0.00	1.97	0.02	2.24	0.00
Inosine	0.80	0.42	0.65	0.05	21.94	0.00	27.01	0.00	24.60	0.00
Glutamine	1.46	0.01	1.20	0.39	1.33	0.09	1.63	0.00	1.41	0.00
Argininosuccinate	0.93	0.70	1.90	0.05	0.70	0.07	0.35	0.00	0.55	0.00
3‐methyl‐2‐oxobutyrate	0.81	0.35	0.36	0.00	62.41	0.00	141.46	0.00	100.82	0.00
3‐methyl‐2‐oxovalerate	0.64	0.07	0.33	0.00	18.95	0.00	36.41	0.00	28.77	0.00
4‐methyl‐2‐oxopentanoate	0.81	0.13	0.34	0.00	43.32	0.00	103.69	0.00	72.49	0.00

### 3.3 Combined analysis of proteomics and metabolomics

#### 3.3.1 The joint pathway analysis

**Figure 8:**
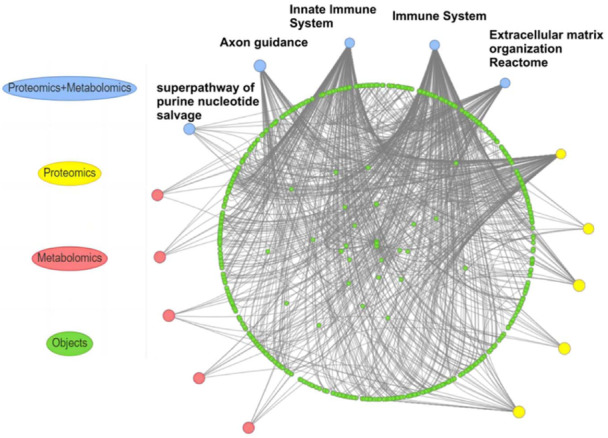
The differential proteins and metabolites joint pathway analysis network diagram.

The joint pathway analysis of differential proteins and metabolites was conducted, the result is shown in Figure 8 and Table 7. As shown, there are five pathway enriched in total. The most enrichedpathways are the immune and innate immune systems.

**Table 7:** The joint pathway analysis of differential proteins and metabolites.

Pathway name	Proteins		Metabolites		*p* joint
	Component ratio	*p* Value	Component ratio	*p* Value	
Immune system	217/725	0.00	3/119	0.79	0.00
Innate immune system	184/725	0.00	3/119	0.63	0.00
Extracellular matrix organization	72/725	0.00	1/119	0.55	0.00
Neutrophil degranulation	116/725	0.00	0	1.00	0.00
Axon guidance	60/725	0.00	1/119	0.48	0.00

#### 3.3.2 The enriched immune related proteins and metabolites

**Figure 9:**
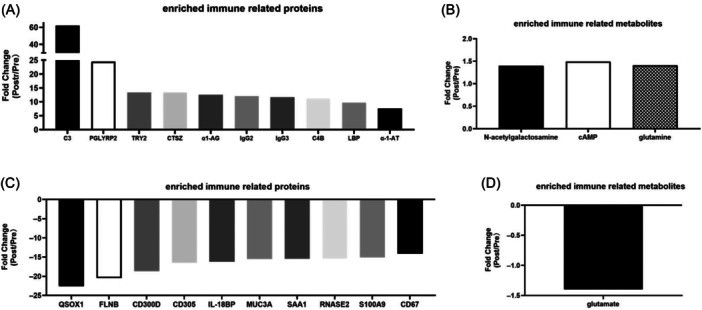
The enriched immune related proteins and metabolites. (A) The upregulated proteins enriched in the immune system include complement 3 (C3), N‐acetylmuramoyl‐l‐alanine amidase (PGLYRP2), trypsin‐2 (PRSS2), cathepsin Z (CTSZ), Al‐pha‐1‐acid glycoprotein 1 (α1‐AG), immunoglobulin heavy constant gamma 2 (IgG2), immunoglobulin heavy constant gamma 3 (IgG3), complement C4‐B (C4B), lipopolysaccharide‐binding protein (LBP), and alpha‐1‐antitrypsin(α−1‐AT). (B) The upregulated metabolites enriched in the immune system are N‐acetylgalactosamine, cAMP, and glutamine. (C) The downregulated proteins enriched in the immune system include sulfhydryl oxidase 1 (QSOX1), filamin‐B (FLNB), CMRF35‐like molecule 2 (CD300D), leukocyte‐associated immuno‐globulin‐like receptor 1 (CD305), interleukin‐18‐binding protein (IL‐18BP), mucin‐3A, amyloid protein‐binding protein 2 (MUC3A), non‐secretory ribonuclease (SAA1), S100A9, and CD67. (D) The downregulated metabolite enriched in the immune system is glutamate.

Results showed that the significantly upregulated proteins are involved to complements, immunoglobulins and to protein catabolism related proteins. Downregulated proteins are related to inflammatory response and protein synthesis. Upregulated metabolites are alpha‐ketoglutarate, cAMP, N‐acetylgalactosamine and downregulated metabolite is glutamate (Figure 9).

### 3.4 Correlation analysis of OPN/CD44 and training loads

**Figure 10:**
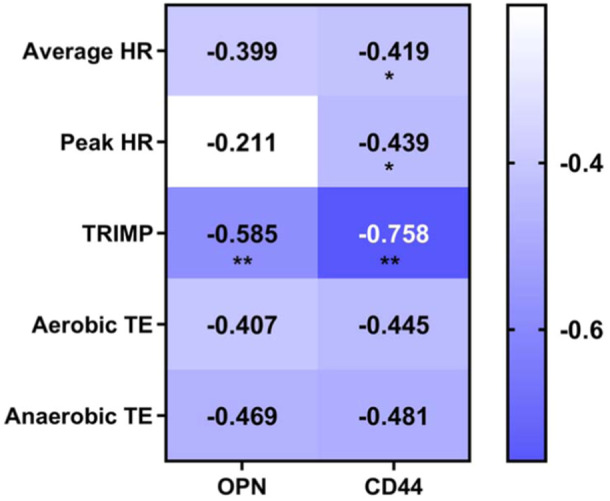
Correlation analysis of the content of OPN/CD44 in urine and training load indicators. The color scale denotes the Spearman's rank correlation coefficient, and the significance levels in the correlation tests are denoted as **p < 0.01. OPN, osteopontin.

Heatmap showing the correlation analysis of the content of OPN/CD44 in urine and training load indicators, in which the TRIMP value is significantly and negatively correlated with the content of OPN/CD44 in urine (Figure 10).

## 4 DISCUSSION

### 4.1 OPN/CD44 is affected by one training course and sex‐dependent

OPN is a phosphorylated glycoprotein initially identified in activated T cells; however, it is also secreted by bone cells, osteoblasts, osteoclasts, activated T cells, and other cells.^30^ OPN plays an important role in inflammatory immunity, osteoblast differentiation, angiogenesis, and steroid hormone response and negatively regulates T cell activation.^31^, ^32^, ^33^ In our study, the OPN levels significantly reduced by nearly 90 times. Total of fourteen sedentary males with overweight or obesity completed two separate two separate exercise trials in randomized and counterbalanced order, two of each trial consisted of a single 30 min workload‐matched bout of either high‐intensity interval exercise (HIIE; alternating 100% and 50% VO_2max_) or continuous moderate intensity (CME; 60% VO_2max_) cycling, OPN were measured pre‐, 0 h post‐, 1 h post‐ and 25 h post exercise. The results showed that the OPN in the blood significantly decreased immediately after exercise and returned to pre exercise levels 25 h after exercise.^34^ CD44 is a widely distributed multifunctional transmembrane glycoprotein on the cell membrane surface and is involved in activating diverse cell types such as immune and epithelial cells and keratinocytes.^35^ Since 1996, the OPN/CD44 pathway has been extensively studied in inflammation, bone remodeling, and other clinical conditions.^36^, ^37^ The OPN/CD44 pathway has also been reported to be associated with signaling molecules such as HIF‐2α and PI3K NF‐κB,^33^, ^38^, ^39^ which can be regulated by exercise. In our study, both OPN and CD44 were enriched in the immune system and were significantly negatively correlated with the training load. However, to date, no specialized research has been conducted on the relationship between exercise and the OPN/CD44 pathway.

Furthermore, our result indicates that there are significant differences in the changes of OPN level in urine caused by exercise between different sexes. Patients with aortic valve stenosis have sexually dimorphic phenotypes, Human female valve leaflets displayed reduced and smaller microcalcifications, but increased OPN expression relative to male leaflets.^40^ A study including of 1452 patients with mild superficial gastritis, atrophic gastritis (AG) or gastric cancer, the levels of serum OPN was higher in men than women.^41^ Our result shows the same trend of change, but it is not significant. a prospective cohort study to assess the independent correlation of 14 urine biomarker with gestational age, sex, and postnatal age in 81 premature infants shows that OPN, were associated with postnatal age and gestational age, but not sex.^42^ The UALCAN database showed that CD44 was independent of sex in gastric cancer but correlated with cancer stage and lymph node metastasis.^43^ The above research results are consistent with our findings in urine, indicating that OPN and CD44 in urine are sex‐specific indicators.

### 4.2 Immune system is affected by one training course and partial sex‐dependent

Immunoactive substances include immunoglobulins, complement proteins, and antibodies. After one training course of 2 h aerobic exercise (average 60% VO_2max_), the significantly unregulated proteins of the STSS, including IgG, IgM, IgA, IgE, C3, C4b, and C9, among others, were classified as classical immune indices. Exercise has a profound effect on the normal immune system.^44^, ^45^, ^46^ Although the research results are inconsistent, IgG levels have been found to be significantly increased in the plasma of endurance athletes. Forty‐four endurance trained participants completed 2 h running at 60% VO_2max_ in 35°C ambient temperature. Blood samples were collected pre‐ and postexercise to determine plasma IgM, IgA, and IgG concentrations. Plasma IgM concentration did not substantially change pre‐ to postexercise, and plasma IgA and IgG increased pre‐ to postexercise.^47^ Eighteen rowers completed a testing course (18 km rowing at 80% of VO_2max_), and the levels of IgG and IgD in serum were found to be increased.^48^ Acute submaximal exercise has the maximum effect on serum IgM levels, which tend to increase.^49^ The effect of exercise on IgE levels remains unclear because individual differences are too large.^50^, ^51^ The content of immunoglobulin in the blood also has sex‐dependent differences. The serum IgG, IgA, IgM, and IgE levels of 110 randomly selected healthy subjects were measured, result shows that the mean IgM level was significantly higher in females than in males, whereas the mean IgA and IgE levels were significantly lower in females.^52^ The above results are consistent with our findings in urine. Although OPN is chemotactic in many cell types, including T and B cells, it enhances B‐lymphocyte immunoglobulin production and proliferation.^53^ Studies have indicated that OPN may inhibit IgE.^54^ Compared with OPN (+/+) mice, significantly increased allergen‐induced IgE levels have been found in OPN (−/−) mice.^55^ Correlation analysis showed that OPN and CD44 were significantly negatively correlated with immunoglobulins.

The complement system, including C1−C9, is another immune pathway that is significantly affected by exercise. A review that included 77 studies and 10,236 participants found that both acute bouts of exercise and exercise training appear to modulate the complement system proteins.^56^ Higher levels of exercise training and cardiorespiratory fitness are commonly associated with reduced C3 in the blood.^56^ The alternative complement system activation pathway is more sensitive to aerobic exercise than to anaerobic exercise training.^57^ Fifty‐one physically active young males performed two types of intensive efforts: aerobic (20‐m shuttle run test) and anaerobic (repeated speed ability test). Venous blood samples were collected before and after each exercise (5 and 60 min) to profile the complement system components. The aerobic effort caused a decrease in the post‐test C3 and increase in post‐test C3a, recovery iC3b, recovery C2 and post‐test C4 levels, while the anaerobic effort caused a decrease only in the post‐test C2 and post‐test C4 levels, an increase in the recovery C3a level.^57^ There are also sex‐differences in the content of some complement proteins. Complement levels and functional activity were determined in 120 healthy volunteers, compared to males, the C3 of females is lower, while factor D concentrations were higher.^58^ The plasma concentration of C5a increased to abnormal levels after a marathon.^59^ Complement activation proteins, including mannan‐binding lectin or fibrin gelatin in plasma, can directly recognize terminal sugar groups, such as N‐acetylglucosamine, mannose, and N‐acetylmannose, on the surface of many pathogenic microorganisms.^60^ We found that N‐acetylgalactosamine (GalNAc) was upregulated and was one of the three enriched metabolites in the joint‐pathway analysis of the immune system. Consistent with our findings, GalNAc‐conjugated RNA interference or interfering RNA therapeutic suppressed the production of C5^61^ and C3^62^ in the liver.

The KEGG pathway for differential metabolites is enriched in glutamine metabolism, arginine biosynthesis, and branched‐chain amino acid metabolism and is mainly related to immunity,^63^ inflammation,^64^ antioxidation,^65^ and myolysis.^66^ Joint‐pathway analysis identified glutamine as the most important immunity‐related metabolite. Glutamine plays multiple roles, including maintaining immune system function,^67^ attenuating exercise‐induced muscle damage,^68^ and regulating glucose/glycogen metabolism^69^, ^70^ during exercise. Some studies have found that plasma glutamine concentrations and glutamine/glutamate ratios are reduced in athletes with chronic fatigue and overtraining syndrome.^71^, ^72^ Aerobic exercise may also increase the glutamine/glutamate ratio. After a 10‐week standardized exercise program, glutamate levels decreased in the serum samples of 37 healthy male participants.^73^ The glutamine/glutamate ratio in the plasma of trained rats was significantly higher than that in sedentary control rats.^74^ In our study, glutamine increased, whereas glutamate decreased, indicating an increase in the glutamine/glutamate ratio. cAMP is another enriched immune‐related metabolite, and the cAMP content increased nearly 2 times in our study. It has been reported that cAMP can increase the glutamine in astrocytes.^75^

## 5 CONCLUSIONS

One training course can lead to the activation of the immune system and a sex‐dependent decrease in the content of OPN. Training load has a significant and negative correlation with the content of OPN and CD44, suggesting that OPN and CD44 could be potential indicators for training load.

According to our research, even a mild aerobic training course can disrupt the immune system, which emphasize the importance of post‐training recovery even after a less intensed training. Meanwhile, it has been proved that OPN is a safe ingredient which has been used in the food industry, for instance, infant formula.^76^ This increase the possibility of a OPN based sports nutrition product development. Sports nutrition professionals including nutritionists may also consider it as an additional supplement to boost the post‐training recovery for athletes.

### 5.1 Limitation of study

Regarding the limitations of the study, due to the small number of elite athletes, the study was conducted on a limited number of participants, resulting in a limited sample size. Another limitation is the lack of standardized diet and drink for the participants. The aim was to avoid any additional stress from changing the diet, so the participants were asked to stick to their daily diet routine.

Even though there are some limitations, our study shows how exercise affects the urine of elite STSS using metabolomics and proteomics. The findings of this study not only provide a theoretical basis for other studies, but also offer novel ideas for developing nutritional products that help recover postexercise fatigue and immune function. Further research should be carried out to examine the effects of different exercise intensities on OPN content in urine and its mechanism through animal and population experiments. As well as the effect of supplementing OPN on exercise recovery.

## AUTHOR CONTRIBUTIONS

**Tieying Li**: Data curation; formal analysis; visualization; writing—original draft; writing—review and editing. **Jing Shao**: Formal analysis; investigation; software. **Nan An**: Methodology; project administration; supervision; writing—review and editing. **Yashan Chang**: Methodology; validation; visualization; writing—review and editing. **Yishi Xia**: Software; writing—review and editing. **Qi Han**: Investigation; methodology. **Fenglin Zhu**: Data curation; formal analysis; investigation; software.

## Data Availability

The data that support the findings of this study are available on request from the corresponding author. The data are not publicly available due to privacy or ethical restrictions.
